# Performance and properties of coking nanofiltration concentrate treatment and membrane fouling mitigation by an Fe(ii)/persulfate-coagulation-ultrafiltration process†

**DOI:** 10.1039/c8ra10094b

**Published:** 2019-05-15

**Authors:** Ming Yang, Jiabin Chen, Boyu Peng, Zhenjiang Yu, Huaqiang Chu, Xuefei Zhou

**Affiliations:** State Key Laboratory of Pollution Control and Resources Reuse, School of Environmental Science and Engineering, Tongji University Shanghai 200092 China zhouxuefei@tongji.edu.cn chq123wd@163.com +86-21-65982693

## Abstract

Coking nanofiltration (NF) concentrates, as typical wastewater with high salinity and refractory organics, have become one of the greatest challenges for “near-zero emission” processes. In our study, an advanced oxidation technology based on ferrous iron/persulfate (Fe(ii)/PS) and polyferric sulfate (PFS) coagulation coupled with ultrafiltration (UF) was used to treat NF concentrates and mitigate membrane fouling. Based on batch experiments, the optimal parameters of Fe(ii)/PS were obtained, during which we discovered that the slow reaction stage of total organic carbon (TOC) removal followed first-order degradation kinetics. Under the optimal reaction conditions, Fe(ii)/PS could efficiently mineralize 69% of organics in coking NF concentrates. In order to eliminate the iron floc generated in the Fe(ii)/PS step, a small amount of PFS (0.05 mM) was added to coagulate the iron floc, which could further improve the effluent quality so that the turbidity, iron content and TOC were significantly reduced by 79.18%, 98% and 21.79% respectively. Gas chromatography coupled with time-of-flight mass spectrometry (GC × GC-TOFMS) and fluorescence excitation-emission matrix spectrometry (EEM) were performed to characterize the removal of phenols, PAHs, quinolines and humic acids in NF concentrates which were responsible for UF membrane fouling. Moreover, scanning electronic microscopy (SEM) and atomic force microscopy (AFM) were conducted to study the surface of the UF membrane after treatment of NF concentrates. The result exhibited that the organic pollutants deposited on the UF membrane surface were reduced by Fe(ii)/PS-PFS pretreatment, and UF membrane flux was thus enhanced. Our results show the potential of the approach of applying Fe(ii)/PS-PFS-UF in NF concentrate treatment.

## Abstract 2

The removal effect of organics and the feasibility of membrane fouling mitigation with Fe(ii)/PS-PFS coupling technology.

## Graphical abstract

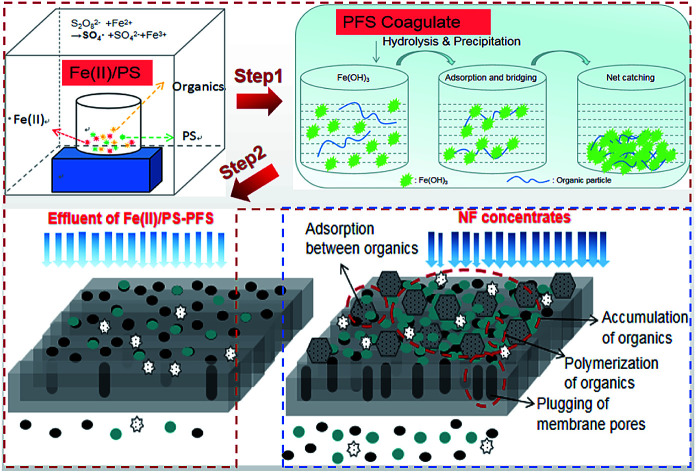

## 1. Introduction

The coal chemical industry is characterized by high water consumption and high organic loads.^1^ Large quantities of coking wastewater with a high content of recalcitrant compounds are produced every year.^2^ To date, “near-zero emission” processes have been developed to reduce wastewater emissions and control the use of fresh water based on the characteristics of the intercepting screening effect and chargeability of NF.^3^ However, the secondarily generated NF concentrates with high salinity and resistant pollutants still need further handling,^4,5^ and the problem of membrane fouling also needs to be addressed.^6^ The control of membrane fouling basically includes three approaches, *i.e.* modification of the membrane,^7,8^ pretreatment of feed water and cleaning of the fouled membrane.^9^ Among these methods, pretreatment of the feed water is the most direct method for industrial use.

At present, coagulation,^10^ adsorption,^11^ and pre-oxidation^12,13^ are commonly applied to NF concentrate pretreatment. However, due to the complex composition of NF concentrates, the treatment efficiency was always quite low by coagulation and adsorption.^14,15^ Besides, these treatment methods would mean extra energy consumption, waste of resources and secondary pollution in the long term.^16^ As for the pre-oxidation approaches, the treatment period was quite long^12^ by the photo-Fenton method. The ozone oxidation method could not be applied to large-scale practice as its strong oxidation corrodes the membrane module,^13^ and might even form byproducts. Instead, there are other pretreatment options such as integrating pretreatment processes to enhance membrane performance and reduce fouling. Integrated pretreatment like ultrafiltration (UF) followed by nanofiltration (NF) comprehensively takes the advantages of each treatment and avoids respective defects^17,18^ which could effectively remove some bacteria, colloids, macromolecular organics,^19,20^ reduce NF membrane fouling and enhance the permeate flux of the system. In addition, the compact process, high degree of automation and stable water quality are regarded by the water treatment industry as the most promising treatment processes.^21,22^ However, the UF approach still faces the same problems that the complex NF concentrates are likely to cause rapid pore blockage. Therefore, finding an efficient, inexpensive, energy-saving resource pretreatment method to further mitigate membrane fouling and prolong membrane life will provide more possibilities for the “near-zero emission” process.

Recently, methods for removing refractory organic pollutants by sulfate radical (SO_4_^−^˙)-based advanced oxidation processes (SR-AOPs) have been widely studied.^23,24^ SR-AOPs have been increasingly applied to degrade organic pollutants such as atrazine,^25^ 4-chlorophenol,^26^ polychlorinated biphenyls,^27^ 2-methylisoborneol and geosmin.^28^ SO_4_^−^˙ can be generated by persulfate (PS) activation with the effect of transition metal ions, ultrasonication, heat or UV radiation.^29^ Fe(ii)/PS (eqn (3)), a nontoxic AOP system with a low cost,^30^ has the potential to be employed into NF concentrates pretreatment. While beneficial, suspended Fe(iii) floc will not be completely precipitated, resulting in adsorption and pore blocking of membrane.^31,32^ Besides, as the membrane pore size is narrowed, various pollutants are deposited on the surface, and thus form a dense fouling layer on the membrane.^33^ Under this condition, coagulation is considered as an indispensable step to be adopted prior to UF to effectively remove the suspended Fe(iii) floc.^34^ In this study, with the effect of low-dose coagulants, the turbidity of effluent, electrical conductivity and the concentration of iron content reduced by 79.18%, 8.83% and more than 98%, respectively. The TOC was further reduced. The results indicated that the preliminary combination of Fe(ii)/PS and PFS was more effective than a single treatment due to the removal of suspended Fe(iii) floc and could control irreversible fouling of the membrane.^35^

Since most of the previous studies used synthetic wastewater in batch experiments,^36,37^ more detailed research is needed to investigate the feasibility of the Fe(ii)/PS system using authentic wastewater. Besides, it is of great importance to explore the optimal conditions for realizing efficient removal of refractory organics from complex real wastewater. The objectives of this study were: (1) to elucidate the efficiency of Fe(ii)/PS-PFS reducing TOC in coking NF concentrates; and (2) to identify the mechanism of organics removal in coking NF concentrates and UF membrane fouling mitigation. Our results are expected to provide a potential pretreatment technology for coking NF concentrates treatment.

## 2. Experiment section

### 2.1 Materials

NF concentrates, which were produced in a double-membrane treatment process (the detailed process is shown in Fig. S1†), were collected from Baogang coking plant, Shanghai, China. The main water quality (raw water) is shown in Table 1.

**Table 1:** The characteristics of the raw water, the effluent of Fe(ii)/PS, the effluent of Fe(ii)/PS-PFS, the permeate of Fe(ii)/PS-PFS-UF and the permeate of raw water

	Raw water	Effluent of Fe(ii)/PS	Effluent of Fe(ii)/PS-PFS	Permeate of Fe(ii)/PS-PFS-UF	Permeate of raw water
pH	7.83 ± 0.21	3.55 ± 0.32	8.62 ± 0.23	8.76 ± 0.21	7.78 ± 0.19
TOC (mg L^−1^)	186.68 ± 2.15	67.22 ± 0.95	52.57 ± 2.13	51.24 ± 0.87	135.85 ± 2.71
Turbidity (NTU)	2.55 ± 0.86	6.34 ± 1.23	1.32 ± 0.43	0.35 ± 0.26	1.56 ± 0.22
Electrical conductivity (mS cm^−1^)	4.86 ± 0.27	6.34 ± 0.95	5.78 ± 0.41	4.76 ± 0.21	4.64 ± 0.31
Chroma (degree)	1680 ± 80	240 ± 80	320 ± 80	160 ± 80	960 ± 80
Fe^2+^ (mg L^−1^)	1.63 ± 0.36	64.52 ± 6.85	0.24 ± 0.02	0.19 ± 0.01	1.61 ± 0.35
Mg^2+^ (mg L^−1^)	2.45 ± 0.35	1.23 ± 0.12	0.86 ± 0.15	0.85 ± 0.05	2.44 ± 0.30
Al^2+^ (mg L^−1^)	0.34 ± 0.11	0.32 ± 0.12	0.21 ± 0.03	0.19 ± 0.01	0.31 ± 0.11
Ca^2+^ (mg L^−1^)	0.54 ± 0.23	0.44 ± 0.16	0.42 ± 0.05	0.34 ± 0.04	0.51 ± 0.20
Cl (mg L^−1^)	0.86 ± 0.18	0.88 ± 0.13	0.78 ± 0.12	0.69 ± 0.12	0.85 ± 0.15

Ferrous sulfate was employed as the activator to generate persulfate radicals. PFS was used as a coagulant. The reagents in this experiment were analyzed pure and purchased from the Sinopharm Chemical Reagent Co., Ltd. The remaining water was prepared from an ultrapure water system (Nanopure Millipore System, D11911, Thermo Scientific). A polyethersulfone (PES) membrane (Microdyn-Nadir, Germany) with pore sizes of 0.05 μm was employed in the filtration experiment. The characteristics of the employed membranes are shown in Table S1.†

### 2.2 Experimental setup

Fe(ii)/PS and PFS coagulation was employed for feed water pretreatment, and retreated water samples were subsequently used for UF membrane filtration.

The oxidation experiments were carried out batch wise in 250 mL glass bottles using 100 mL coking NF concentrates. During this step, the pH of the solution was adjusted to 3.0.^38,39^ The samples were shaken at 200 rpm. PS was added from 0 mM to 4 mM of the stoichiometric dose,^40^ Fe(ii) was added from 0 mM to 6 mM of the stoichiometric dose, and the reaction was maintained for 2 h. Different reaction times were maintained to examine the effect of the initial PS dose and the initial Fe(ii) dose on the Fe(ii)/PS process.

PFS of 0.5 mM was added into 100 mL of the oxidizing effluent to start the coagulation reaction. The pretreatments were performed with rapid mixing for 1 min at 200 rpm, followed by slow mixing for 20 min at 50 rpm. Then, the samples were taken, filtered and immediately analyzed. All of the experiments were conducted in triplicate. The results shown are the averages of the duplicates.

The filtration experiment was performed in filtration cells in the dead-end mode (details are shown in Fig. S2†). The PES membrane filtration was conducted in a filtration cell (MSC300, Mosu Science Equipment, Shanghai) with an effective volume of 300 mL. A nitrogen gas bottle connected to the filtration cell was used to maintain a constant transmembrane pressure of 100 kPa. UF membranes were placed at the bottom of the filtration cell during the experiment, and each filtration test was run for three cycles.

### 2.3 Analytical method

pH was measured by a pH meter. A Hach 2100N turbidimeter (Loveland, CO) was used for the turbidity analyses. Electrical conductivity was obtained by a conductivity meter. A Hach DR-2800 spectrophotometer was used for the colorimetric analyses. The analysis was carried out using either inductive coupled plasma emission spectrometer (ICP). Chloride concentrations was determined using potentiometric titration methods. The hydrophilicity/hydrophobicity of the membranes was indicated by measuring their water contact angles using a water contact angle analyzer (Dataphysics OCA 15EC, DE). The membrane porosity *ε* (%) was calculated by a gravimetric method^41^ and mean pore size *r*_m_ (nm) was determined using the Guerout–Elford–Ferry equation^41,42^ based on the data of porosity and pure water flux. Zeta potential was measured by a Zeta Potential Analyzer (Zetasizer Nano ZS 90, UK). The dissolved organic carbon (DOC) concentration (after 0.45 μm membrane filtration) of the water samples was determined using a TOC analyzer (Multi N/C 2100, JENA). The TOC degradation efficiency was calculated as follows:1
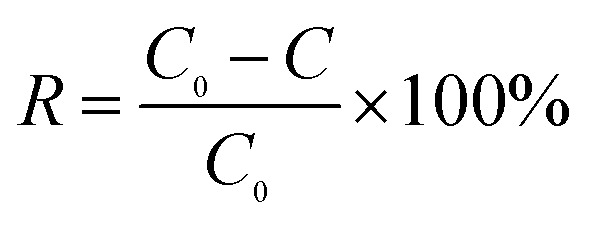
In eqn (1), *C*_0_ = TOC concentration before oxidation, mg L; *C* = TOC concentration after oxidation, mg L^−1^.

EEM was used to characterize the fluorescent components in the water. The EEM spectra were generated using a fluorescence spectrophotometer (F4600, Hitachi, Japan) with excitation (Ex) wavelengths of 200–550 nm and emission (Em) wavelengths of 300–650 nm. The EEM spectrum of ultrapure water was subtracted from each EEM sample to remove most of the Raman scatter peaks.^43^

Molecular distribution was determined by Gel Permeation Chromatography (GPC), Agilent Technologies, USA (TSK gel: G3000PWXL; column no. S0127; temperature: 40 °C; detector: UV254).

Refractory organic pollutants were analyzed using a Pegasus 4D GC × GC-TOFMS instrument (LECO Corp., St. Joseph, MI, USA) to detect compounds in complex samples. This system utilizes a fixed quad-jet modulator consisting of two liquid nitrogen jets and two pulsed hot-air jets to trap and refocus compounds eluted from the first dimension (1D) column.^44^ The modulation period was 2.5 s. The hot pulse duration was 0.60 s. Helium was used as the carrier gas at a constant flow rate of 1 mL min^−1^. The mass spectrometry (MS) transfer line temperature was 330 °C. Ionization was performed with an electron ionization (EI) energy of 70 eV and an ion source temperature of 250 °C. The collected mass range was 50–550 amu with an acquisition rate of 200 scans per s after a solvent delay of 450 s.^45^

Attenuated total reflectance-Fourier transform infrared spectroscopy (ATR-FTIR) (Nicolet 8700, Thermo Electron Corporation, USA) with a resolution of 4 cm^−1^ in the range of 400–4000 cm^−1^ was used to obtain information about the functional groups of the membrane surfaces. Membrane samples were held between the diamond plate and the pressure column with the separation layer facing the beam.

The surface and cross-section of the membranes were visualized by SEM imaging. Cross-sectional samples were prepared by mechanically fracturing the membrane in liquid nitrogen. These samples were then dried and sputter coated with a 5 nm thick Au/Pd layer under an argon atmosphere to obtain the necessary conductivity. Microscopic analyses were performed at 12 kV using a Phenom proX SEM-EDS (the USA).

The surface roughness of the membranes was investigated by atomic force microscopy (AFM, CSPM5500) with a non-contact mode. Roughness parameters such as root-mean-square roughness (*R*_q_), mean roughness (*R*_a_) and maximum roughness (*R*_z_) were quantified from the topography images of the 10 μm × 10 μm area.

### 2.4 Filtration performances

Water flux was carried out at 25 °C in a filtration cell (MSC300, Mosu Science Equipment, Shanghai), with an effective test area of 36 cm^2^. An electronic balance is linked to a computer to automatically log weight data every 5 s. The permeation flux (*J*) was calculated using the following equation:^46^2
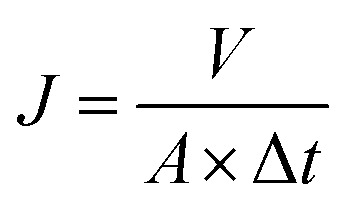
where *V* is the permeate volume (L), *A* is the effective membrane area (m^2^), and Δ*t* is the permeation time (h).

## 3. Results and discussion

### 3.1 The optimization of the Fe(ii)/PS-PFS process parameters

#### 3.1.1 The effect of PS and Fe(ii) dose

**Fig. 1:**
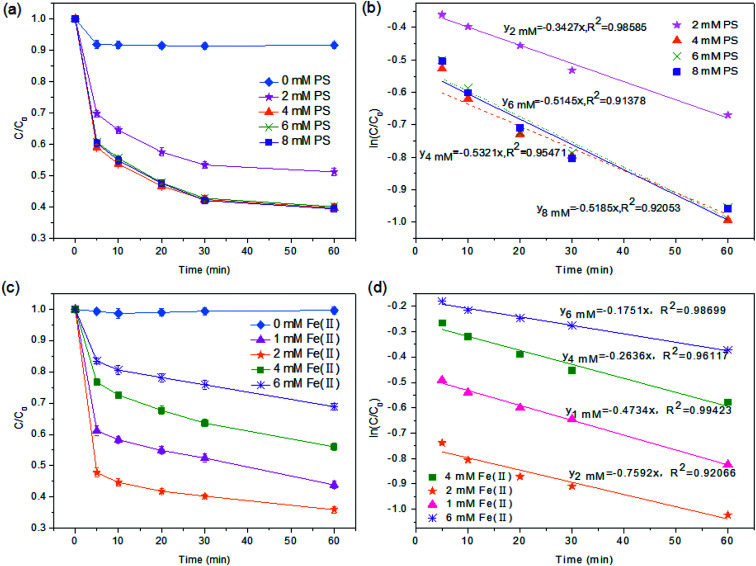
Compatibility of the activated methods. (a) Degradation rate and (b) the first order kinetic equation (varying conditions are based on the control experiment, pH = 3.0, [Fe(ii)]_0_ = 2 mM and *T* = 25 °C). Effect of the Fe(ii) initial dose. (c) Degradation rate and (d) the first order kinetic equation (varying conditions are based on the control experiment: pH = 3.0, [PS)]_0_ = 4 mM and *T* = 25 °C). *C*_0_ represents the initial concentration of NF concentrates while *C* represents the concentration of TOC after Fe(ii)/PS treatment.

As a SO_4_^2−^ source to the Fe(ii)/PS reaction, PS dosage is of great importance for TOC degradation. The effect of PS dosage was examined by varying the PS/Fe(ii) ratios from 1 : 1 to 4 : 1. As shown in Fig. 1a, in the condition of [Fe(ii)] = 2 mM and [PS] = 0 mM, the TOC degradation efficiency was only 8.17%. When the dosage of PS was increased from 2 to 4 mM, *i.e.* the [PS]/[Fe(ii)] ratio was increased from 1 : 1 to 2 : 1, the TOC degradation increased significantly from 49.79% to 62.99% within 60 min. However, a further increase in the PS dosage only caused a slight increase in the TOC mineralization. For example, the efficiency of TOC mineralization at 60 min only achieved 63%, while the ratio of [PS]/[Fe(ii)] was increased to 4 : 1. In addition, Fe(ii) could cause the coking NF concentrates to develop colloidal properties like the Fe(iii) floc under alkaline conditions which could lead to the coagulation and removal of the organic pollutants. When the pH of the solution was adjusted to 3.0 (Section 2.2), Fe(ii) exists in the form of hydrates such as [Fe(H_2_O)_6_]^2+^, [Fe(H_2_O)_6_]^3+^, and [Fe(H_2_O)_5_]^2+^ (eqn (5) and (6)) which were unfavorable for coagulation.^47^ Therefore, excessive amounts of PS could not improve the mineralization of TOC, which was attributed to the fact that SO_4_^−^˙ might react with excess PS to produce a persulfate radical (S_2_O_8_^−^˙) with an oxidizing power lower than SO_4_^−^ (eqn (9)).^48^ This is consistent with the observations made by other researchers.^49,50^

Based on the fact that Fe(ii) can catalyze PS to produce SO_4_^−^˙ radicals,^51^ the significance of Fe(ii) dosage for TOC degradation should be taken into consideration. The effect of Fe(ii) dosage on TOC mineralization with different molar ratios of [PS]/[Fe(ii)] was shown in Fig. 1c. When the Fe(ii) concentration varied from 1 to 2 mM ([PS]/[Fe(ii)] = 4 : 1, 2 : 1), the efficiency of TOC degradation at 60 min was 38% and 64%, respectively. However, with a further increase of Fe(ii) dosage up to 6 mM ([PS]/[Fe(ii)] = 1 : 1.5), the efficiency of TOC mineralization reduced to 31%. This suggested that excessive amounts of Fe(ii) consumed SO_4_^−^˙ (eqn (4)) and thus decreased the concentration of SO_4_^−^ in the system. Meanwhile, a high concentration of SO_4_^−^˙ resulted in the occurrence of a self-quenching reaction^52^ (eqn (8)) for NF concentrates. Similar results have been reported in other studies.^53^ Therefore, it could be concluded that while a moderate amount of Fe(ii) is required to effectively activate PS to generate a sufficient amount of SO_4_^−^˙ in the reaction solution, an excessive amount of Fe(ii) is detrimental to the TOC mineralization.

3 S_2_O_8_^2−^ + Fe^2+^ → SO_4_^−^˙ + SO_4_^2−^ + Fe^3+^

4 SO_4_^−^˙ + Fe^2+^ → SO_4_^2−^ + Fe^3+^

5 Fe^2+^ + 6H_2_O + H^+^ → [Fe(H_2_O)_6_]^3+^

6 Fe^2+^ + 5H_2_O → [Fe(H_2_O)_5_]^2+^

7 H^+^ + S_2_O_8_^2−^ → HS_2_O_8_^−^

8 SO_4_^−^˙ + SO_4_^−^˙ → S_2_O_8_^2−^

9 SO_4_^−^˙ + S_2_O_8_^2−^ → S_2_O_8_^−^˙ + SO_4_^2−^

To better evaluate the process parameters for pretreatment performance, TOC-based kinetic analysis has been conducted to understand the mineralization behavior of organic pollutants.^54,55^ The following rate equation has been applied to describe the kinetics of TOC mineralization in the process.10
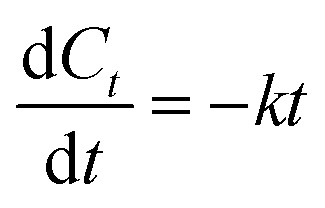
In eqn (10), *C*_*t*_ = the concentration of TOC at the oxidation time, mg L; *K* = reaction rate constant; *T* = reaction time.

As shown in Fig. 1b and d, the ln(*C*_0_/*C*)–*t* plot showed the TOC degradation at different PS concentrations and Fe(ii) concentrations, respectively. And the results in Fig. 1a indicated that TOC removal could be divided into two stages: the rapid reaction stage and the slow reaction stage. In the first 5 min, the main reaction happening in the solution was eqn (3), and the rapidly generated SO_4_^−^˙ was used for the mineralization of TOC. After 5 min of reaction, Fe(ii) mainly changed to Fe(iii) by eqn (4). Under this condition, TOC removal in the solution was relatively stable, and the plot of (ln(*C*_0_/*C*)) *versus* time (inset of Fig. 1b) showed a linear relationship, indicating that the TOC degradation followed the first-order kinetic model.

The results of the series of kinetics experiments under various conditions (*i.e.*, different PS concentrations and different Fe(ii) concentrations) are presented in Fig. 1b and d. According to the pseudo first-order of TOC degradation, when [PS] was 2 mM, 4 mM, 6 mM and 8 mM, the degradation rates were 0.3427, 0.5321, 0.5145 and 0.5185 min^−1^, respectively (pH = 3.0, [Fe(ii)] = 2 mM). It appears that when [PS] was 4 mM, the TOC degradation rate by PS/Fe(ii) process was the fastest. Meanwhile, the pseudo first-order rate constants of TOC degradation under pH = 3.0, [PS] = 4 mM with [Fe(ii)] = 1 mM, 2 mM, 4 mM and 6 mM were 0.47, 0.76, 0.26 and 0.18 min^−1^, respectively (Fig. 1d). And the fastest rate of TOC degradation appeared when [Fe(ii)] = 2 mM. The result was compatible with the conclusions of Liang *et al.*^38,39^ Hence, the technology of Fe(ii)/PS pretreatment is effective to remove organics in the coking NF concentrates under the optimal experimental conditions ([PS] = 4 mM and [Fe(ii)] = 2 mM, pH = 3.0).

#### 3.1.2 The effect of PES for coagulation

The range of important parameters for water quality monitoring is summarized in Table 1. Although the TOC of the oxidizing effluent of Fe(ii)/PS was significantly reduced compared with NF concentrates, high turbidity, electrical conductivity and iron content still existed in the solution. Thus, the coagulation process was conducted. As a result, the turbidity and electrical conductivity of the coagulation effluent substantially reduced by 79.18% and 8.83%, respectively, in comparison to the oxidizing effluent of Fe(ii)/PS. The TOC in the effluent further reduced by 21.79%, and the final iron content was 0.24 ± 0.02 mg L^−1^, being reduced by more than 98%. The combination of Fe(ii)/PS and PFS was more effective than a single treatment and could compensate for the single Fe(ii)/PS process by effective removal of suspended Fe(iii) floc with the addition of a low-dose coagulant, which can further improve the quality of product water, mitigate pore blocking and form a cake layer. Among the selected coagulants,^34^ Wu *et al.*^56^ concluded that PFS was the most effective in controlling membrane fouling. With the final Fe(ii) concentration reducing to 0.3 mg L^−1^, no membrane fouling occurred. Moreover, the formation of flocs with good permeability on the membrane surface was beneficial to mitigating the fouling during the coagulation-ultrafiltration process.^47^ So coagulation is considered a destabilization process of colloidal particles.^57,58^

### 3.2 The removal performance of organics under optimal Fe(ii)/PS-PFS conditions

#### 3.2.1 The removal performance of macromolecular organics

**Fig. 2:**
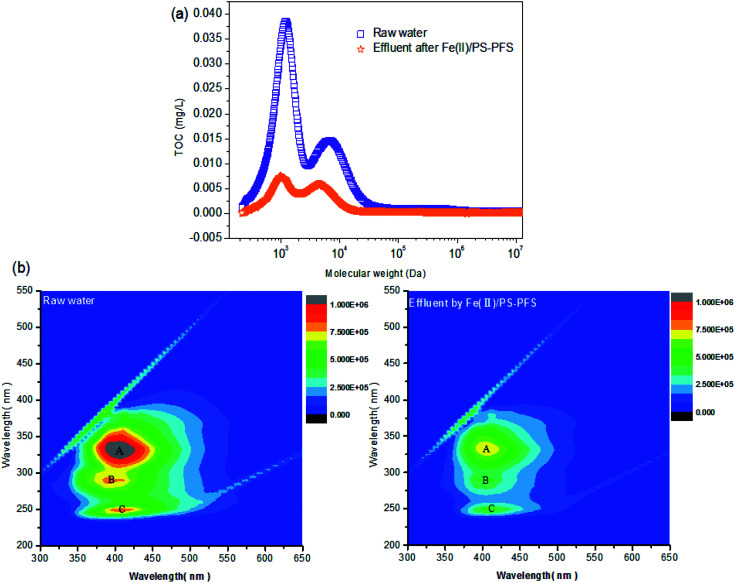
Compatibility of the Fe(ii)/PS processes experimental data based on macromolecular organics. (a) Gel permeation chromatography analysis and (b) three-dimensional fluorescence excitation-emission matrix spectrometry (3D-EEM) analysis (pH = 3.0, [PS]_0_ = 4 mM, [Fe(ii) ]_0_ = 2 mM and *T* = 25 °C).

GPC and EEM characterizations were applied to detect the removal of macromolecular organic pollutants under optimal conditions (pH = 3.0, [PS] = 4 mM and [Fe(ii)] = 2 mM, PFS = 0.5 mM). In Fig. 2a, the MW distribution of the NF concentrates is shown, which implied that biopolymers (MW > 10 kDa), humic substances and other small MW organic pollutants are presented in raw water. As expected, extensive organic pollutants over a wide MW range have been removed in comparison to the effluent after coagulation. The results indicated that the Fe(ii)/PS-PFS coupling process achieved substantial removal of organic pollutants in the MW range between 1 kDa and 10 kDa. The fluorescence EEM spectra of organics in different systems are presented in Fig. 2b. The peaks A (Ex/Em 330/400 nm), B (Ex/Em 280/390 nm) and C (Ex/Em 250/410 nm) exhibited high intensities in the EEM spectra which were associated with humic-like substances^59^ and soluble microbial byproduct-like substances (SMP-like). A SMP-like substance is defined as a soluble extracellular polymeric substance (EPS), mainly containing small carbonaceous compounds derived from the original substrate^60^ and protein-like substances.^59^ The intensity of the fluorescence peaks, especially peak A followed the intensive sequence of the raw water > the effluent after Fe(ii)/PS-PFS pretreatment, indicating that the coupling process could remove macromolecular organics such as humic-like, SMP-like and protein-like substances in coking NF concentrates. The result was similar to the previous chapter (Section 3.1).

#### 3.2.2 The removal performance of refractory organics

**Fig. 3:**
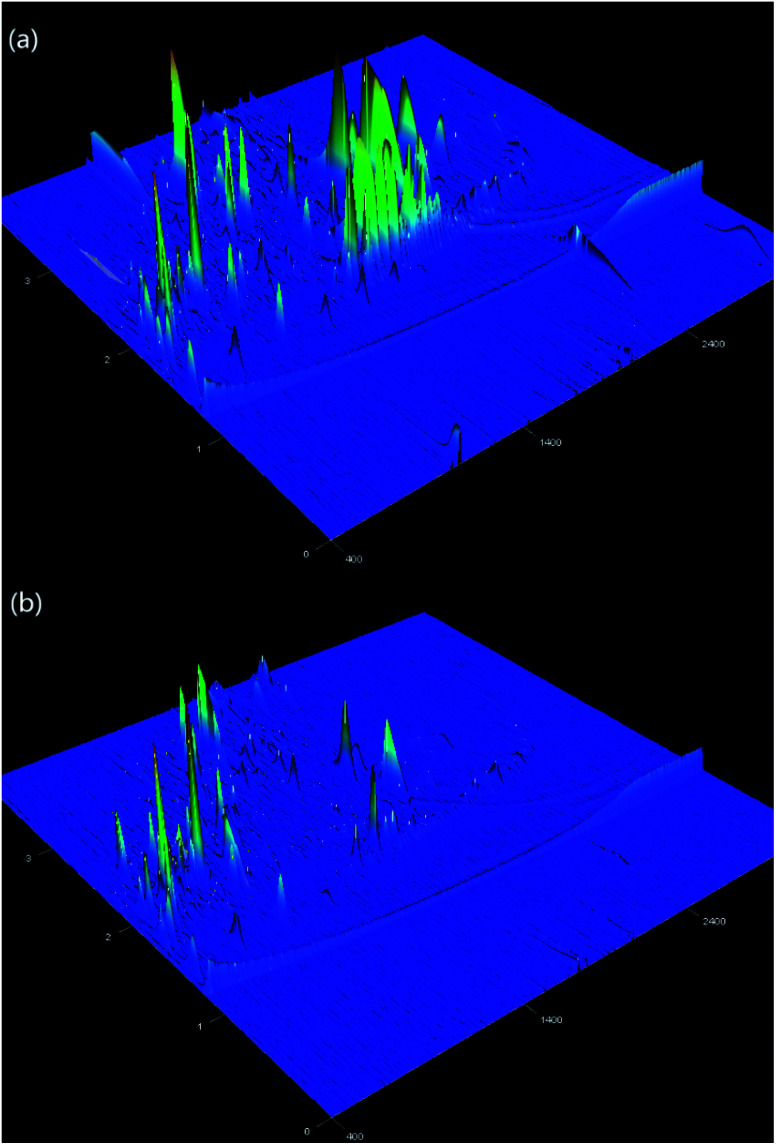
Compatibility of the Fe(ii)/PS processes experimental data based on refractory organics. Pegasus 4D two dimensional gas chromatography-mass spectrometry (GC × GC-TOFMS) analysis. (a) Raw water and (b) effluent by Fe(ii)/PS-PFS (pH = 3.0, [PS]_0_ = 4 mM, [Fe(ii) ]_0_ = 2 mM and *T* = 25 °C).

GC × GC-TOFMS was applied to detect the removal of refractory organic pollutants under optimal conditions (pH = 3.0, [PS] = 4 mM and [Fe(ii)] = 2 mM, PFS = 0.5 mM). The result is shown in Fig. 3. Refractory organic pollutants of 22 and 243 were measured, accounting for 80.69% and 14.96% of the total area, respectively.^44,61^ After Fe(ii)/PS-PFS pretreatment, the aggregate area reduced by 76.27%, and the TOC removal efficiency was the same as that with the Fe(ii)/PS-PFS coupling process. At the same time, the peak area of seven principal organic pollutants (shown in Table S2†) were listed, which revealed several refractory organics, such as phenol, *p*-nitrophenol, indole, methylquinoline, polycyclic aromatic hydrocarbons (PAHs) and a small amount of butanones, butenals and phthalates. Among them, phenol, PAHs and quinolines were the main constituent substances of TOC, which had a high concentration in the coking wastewater.^62^Fig. 3b showed that the intensities of refractory organics were much weaker in the effluent, indicating that the organic pollutants with complex molecular structures experienced open-loop and chain-breaking oxidation by Fe(ii)/PS-PFS. Thus, we could draw the conclusion that refractory organics such as phenols, PAHs, quinolines and humic acids could be effectively degraded under optimal conditions by Fe(ii)/PS-PFS coupling technology.

### 3.3 Fe(ii)/PS-PFS-UF process

Table 1 summarizes the relevant parameters of the water quality. Although the TOC of the permeate of raw water was slightly reduced compared with NF concentrates, there was also high TOC, turbidity, electrical and conductivity. Apparently, compared with the oxidizing effluent of Fe(ii)/PS and PFS coagulation, the turbidity of the permeate of UF substantially reduced by 86.27%, the electrical conductivity substantially reduced by 8.83% with TOC reducing by 72.55%, and iron content was 0.19 ± 0.02 mg L^−1^, reduced by more than 98%. As mentioned previously, the preliminary combination of Fe(ii)/PS and PFS was shown to be effective for the UF process. To comprehensively assess the performance of the Fe(ii)/PS-PFS-UF system, essential characterizations are necessary to elucidate the changes of the UF membrane in the reaction process.

#### 3.3.1 Investigation of the chemical composition on the membrane surface

**Fig. 4:**
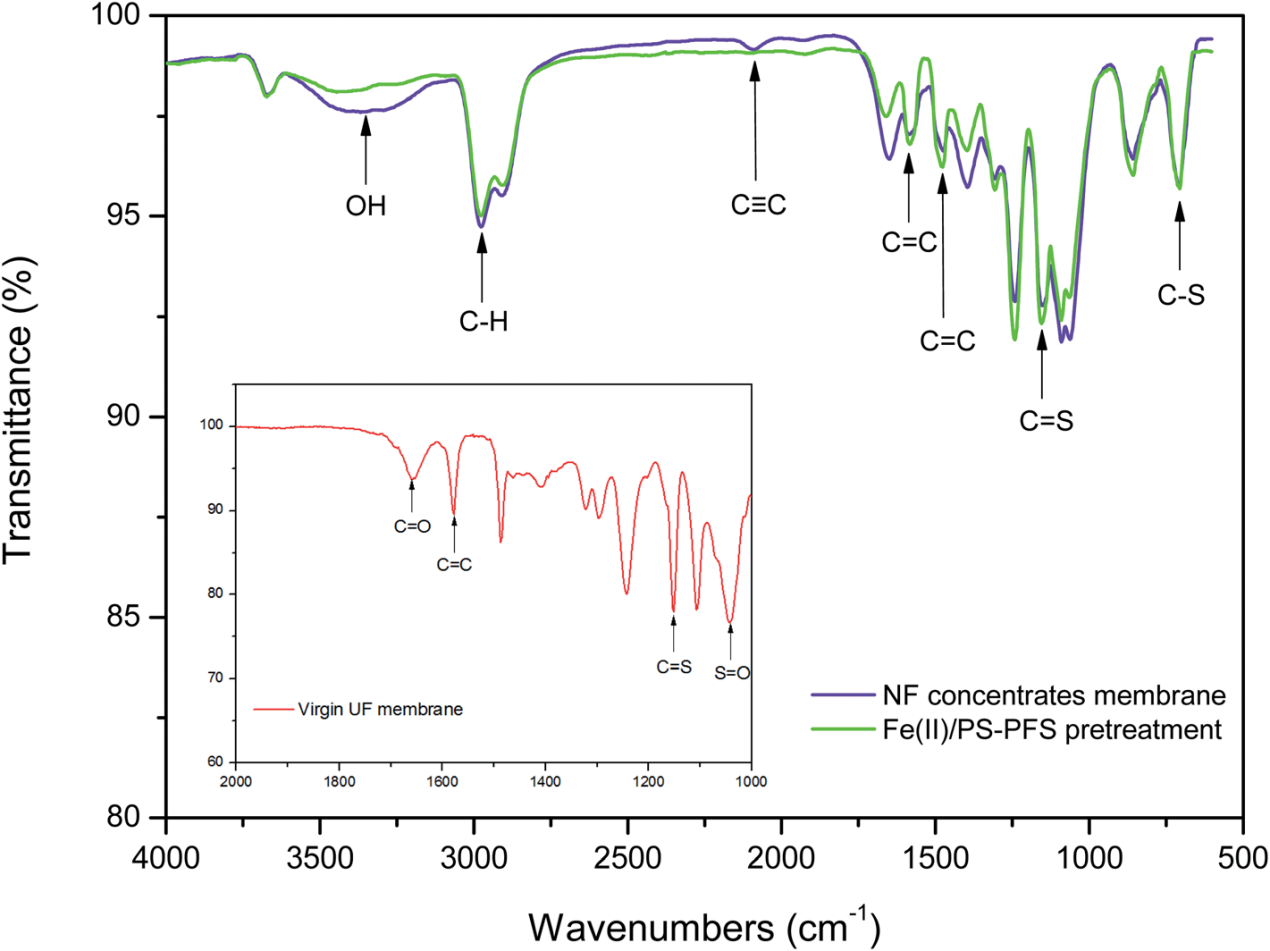
Attenuated total reflectance-Fourier transform infrared spectroscopy (ATR-FTIR) analysis. The red line refers to the virgin UF membrane, the purple line refers to NF concentrates membrane and the green line refers to Fe(ii)/PS-PFS pretreatment (pH = 3.0, [PS]_0_ = 4 mM, [Fe(ii) ]_0_ = 2 mM and *T* = 25 °C). The virgin UF membrane (inserted figure) indicates signals assigned to the PES membrane.

FTIR analysis was applied to analyse the nature of the organics on membranes.^63^ The spectra of the virgin membrane and two kinds of fouled UF membranes are presented in Fig. 4. The spectrum of the virgin membrane was similar to the specific ATR-FTIR absorbance peaks typical for PES membranes.^64^ However, the decrease in the absorbance intensity of typical peaks, and the appearance of additional peaks both suggested that the membranes were severely fouled. Based on Fig. 3, the spectra of the two kinds of fouled UF membranes had similar peaks. Generally, there were four distinct absorption peaks in the picture. The main absorption bands were in the range of 3440–3245 cm^−1^ (–OH stretching), which indicated that both raw water and effluent contained –OH (alcohol or phenol, most likely phenolic hydroxyl groups). The difference was that the raw water has a weak absorption peak, which was the unsaturated double bond (C=C) and the triple bond (C≡C) stretching vibration in the range of 2500–1900 cm^−1^. The results indicated that there were double bonds, aromatic and other compounds which contained methyl groups and alkyl groups in the raw water and the effluent from the coking wastewater. This was consistent with the analysis results of phenols, quinolines and other organic compounds detected by GC × GC. Moreover, compared with the membranes of NF concentrates, the peaks of the membranes after Fe(ii)/PS-PFS pretreatment decreased in both the absorbance intensity and peak quantity, which suggested that the membrane fouling could be addressed.

#### 3.3.2 Investigation of the membrane morphology and structure

**Fig. 5:**
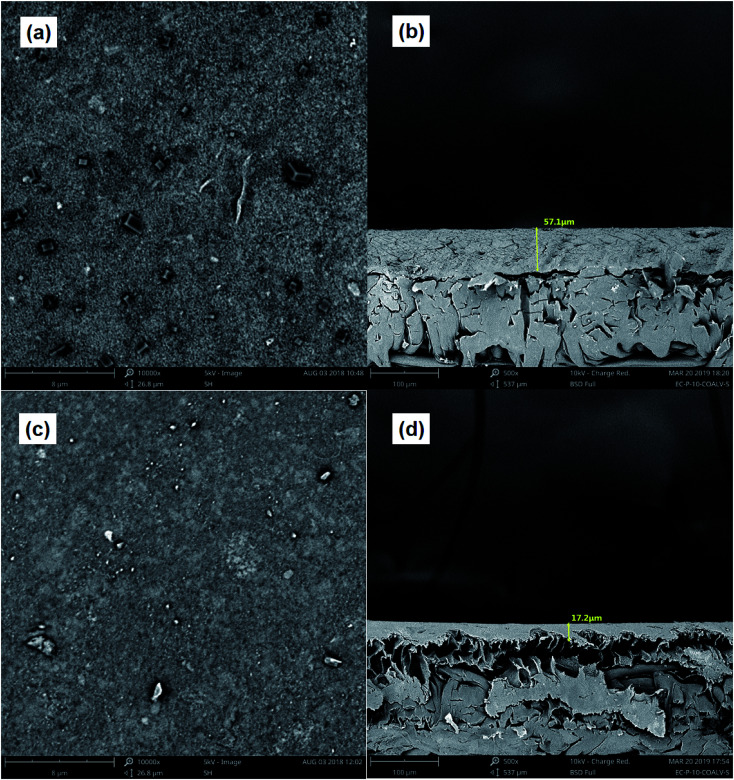
SEM images of membranes. (a) Surface without pretreatment (10k×), (b) cross-section without pretreatment (5k×), (c) surface with pretreatment (10k×) and (d) cross-section with pretreatment (5k×).

In order to investigate the effect of Fe(ii)/PS-PFS pretreatment on the microstructure of the membranes, SEM micrographs and AFM analysis of PES membranes with different feed water have been obtained. The SEM images are displayed in Fig. 5. When pollutants were filtrated through the UF membrane after Fe(ii)/PS-PFS pretreatment, there was a cake layer on the membrane surface. Some pores remained on the membrane surface due to the smaller molecular weights of pollutants to the pores diameter of UF membrane, such as dissolved organic and inorganic ions^65^ (Fig. 5a). Meanwhile, Fig. 5b exhibits the cross-section after filtration, the thickness of which was approximately 57.1 μm. Additionally, when pollutants were filtrated through the UF membrane without pretreatment, the membranes were irregular and rough, and cake layers were deposited on the surface of the membranes, including large particles and some foulants^66^ (Fig. 5c). Besides, Fig. 5d showed the cross-section after filtration, the thickness of which was approximately 17.2 μm. However, compared with the membrane of the Fe(ii)/PS-PFS-UF process, the parallel UF membrane surface seemed to be denser and more compact, and more organics were deposited, indicating the addition of PFS can reduce the deposition of foulants on the membrane surface.

**Fig. 6:**
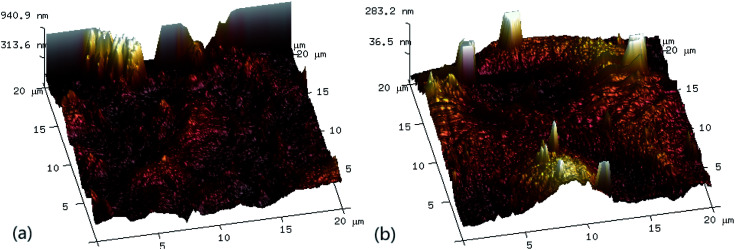
AFM three-dimensional surface images of membranes. (a) Surface without pretreatment and (b) surface with pretreatment.

Fig. 6 shows the three-dimensional surface AFM images of the UF membrane surfaces. The roughness parameters of the surfaces of the UF membranes are given in Table 2. Roughness parameters could be obtained with the AFM analysis software. There exists mean roughness (*R*_a_), root mean square of *Z* data (*R*_q_) and maximum roughness (*R*_z_). It was observed that the roughness parameters of the NF concentrates membranes were larger than the membrane with the effluent of Fe(ii)/PS-PFS (shown in Table 2). The *R*_a_ value decreased from 85.7 nm (NF concentrates) to 36.4 nm (effluent of Fe(ii)/PS-PFS), which was possibly due to the large particles and more organic foulants from NF concentrates within the membrane surface. Xu *et al.*^67^ reported that a membrane with smoother surface possesses greater antifouling capability.^68,69^ Therefore, the membranes with Fe(ii)/PS-PFS pretreatment turned out to have a potential antifouling tendency, which was consistent with the flux recovery results of the membranes depicted in the later part. This may be caused by the degradation and transformation of the macro-molecular and refractory organics after Fe(ii)/PS-PFS pretreatment. In addition, pollutants and ferric colloids after advanced oxidation in the solution aggregated into large particles and precipitated by flocculation of PFS (Table 1). Coagulants were considered to change the particle-size distribution of organic matters in the feed towards larger fractions, with a notable reduction in colloidal matter.^70^ Therefore, Fe(ii)/PS-PFS coupling technology can be used to improve membrane fouling.

**Table 2:** Porosity, surface mean pore size and roughness parameters of the membranes

Membranes	Mean pore size (nm)	Roughness		
		*R*_a_ (nm)	*R*_q_ (nm)	*R*_max_ (nm)
PES-raw water	0.05	85.7	124.0	1770.0
PES-effluent of Fe(ii)/PS-PFS	0.05	36.4	53.6	1210

#### 3.3.3 Investigation of the membrane performances

**Fig. 7:**
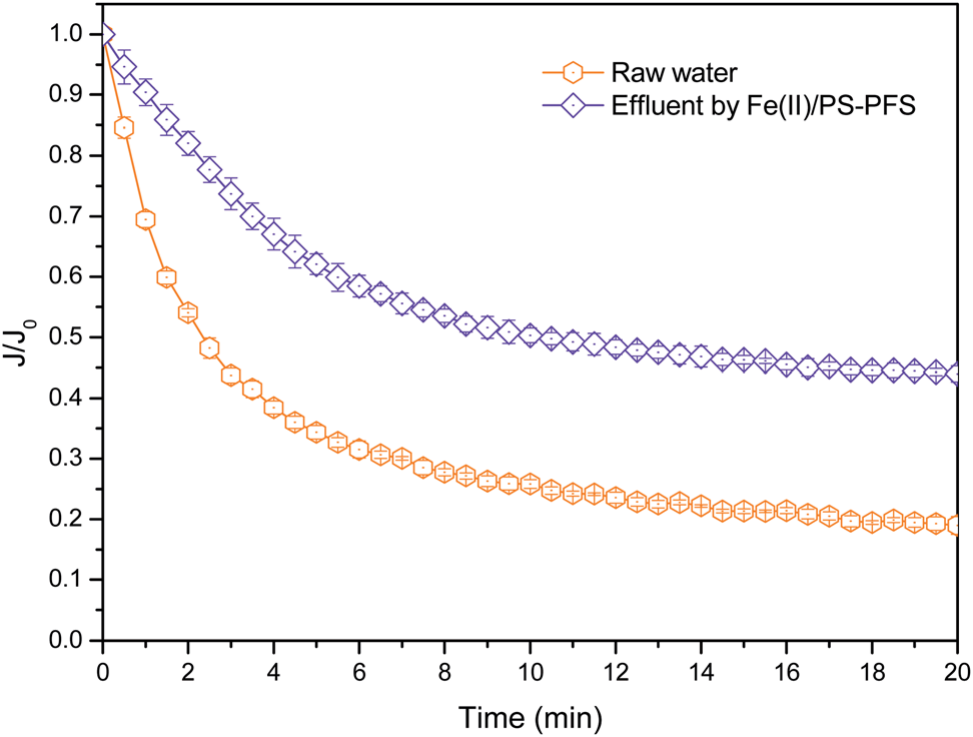
Effects of Fe(ii)/PS on the membrane performance. Flux decline with different feed water. *J*_0_ represents the initial permeation flux of pure water while *J* represents the permeation flux of NF concentrates.

The flux curve profiles during the filtration of coking NF concentrates are shown in Fig. 7. As for the NF concentrates, the flux substantially decreased in the initial filtration phase and subsequently reached a plateau, and the specific flux was finally reduced to 0.185. When Fe(ii)/PS-PFS was added to the feed water, the flux decline during the filtration was slightly alleviated, and a specific flux of 0.438 was obtained at the end of filtration. The reasons why Fe(ii)/PS-PFS techniques could effectively maintain the flux are as follows. First, this may be caused by the influence of organics in the NF concentrates.^71^ The degradation and transformation of the refractory organics after Fe(ii)/PS-PFS pretreatment can mitigate the flux decline (Fig. S5† mechanism of Fe(ii)/PS-PFS). Second, this may be due to the reduction of macromolecule organics such as humic-like, SMP-like and protein-like substances by Fe(ii)/PS-PFS pretreatment. Yuan^72^ studied the pollution of humic acid to the hydrophilic ultrafiltration membrane. It was found that the adsorption and deposition of humic acid on the surface of the membrane could cause serious membrane fouling. Third, Fe(iii) was generated during Fe(ii)/PS oxidation, and the coagulation effect of Fe(iii) and PFS could be utilized for membrane fouling control and to improve the cake layer structure.^73^ Yu *et al.*^74^ demonstrated that Fe(iii) caused natural organic matter to aggregate and form large flocs, lowering the thickness and density of the cake layer during filtration.

**Fig. 8:**
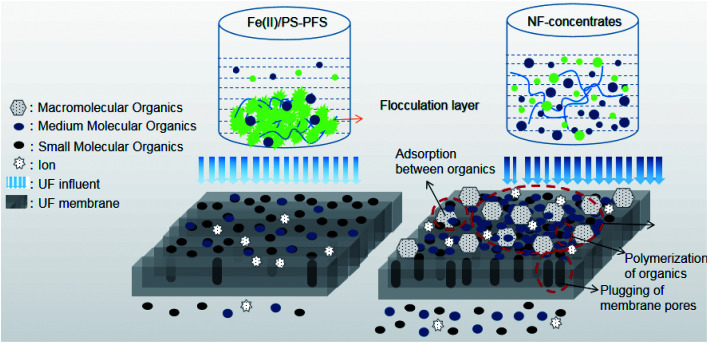
Effect of Fe(ii)/PS-PFS on mitigation of membrane fouling.

In order to further explain the treatment process, the forming fouling mechanism of the membrane is shown in Fig. 8. The shape and structure of the obtained surface greatly depended on organic pollutants such as phenol, quinoline and humic acid from NF concentrates deposited on the membrane surface. With the high concentration of organic pollutants in the NF concentrates, substantial organics were attached to the membrane surface after filtration. These organics tended to accumulate at the membrane pores and block the pores. Then, a new layer was gradually generated with an increasing amount of organics deposited on the membrane surface, creating a strong resistance to water permeation. Therefore, organics were probably the reason for the antifouling property of the Fe(ii)/PS-PFS pretreatment. As an effective pretreatment method, Fe(ii)/PS-PFS can be effective in improving the permeability for the mitigation of membrane fouling.

## 4. Conclusions

This study proposed a Fe(ii)/PS-PFS coupling technology to achieve both the efficient removal of organics in raw water and the mitigation of membrane fouling. The effect of PS concentration and Fe(ii) concentration for TOC degradation and its dynamics were analyzed. The optimal experimental parameters of Fe(ii)/PS pretreatment technology are confirmed ([PS] = 4 mM and [Fe(ii)] = 2 mM, and pH = 3.0). In addition, the quality of the treated water and membrane fouling can be efficiently improved by added 0.5 mM PFS. Organic pollutants deposited on the membrane surface and plugged in the membrane pores were obviously reduced with the transformation of refractory organics and macromolecule organics. It is worth mentioning that membrane flux was also significantly improved, further confirming that the flux declining could be effectively mitigated by Fe(ii)/PS-PFS coupling technology. As an effective pretreatment method, Fe(ii)/PS-PFS exhibits good performance in the mitigation of membrane fouling, which may have great potential in NF concentrate treatment.

## Conflicts of interest

There are no conflicts to declare.

## Supplementary Material

RA-009-C8RA10094B-s001
