# Supplementary material for: Performance and properties of coking nanofiltration concentrate treatment and membrane fouling mitigation by an Fe(ii)/persulfate-coagulation-ultrafiltration process
Source: RSC Adv. 2019 May 15;9(27):15277–87. doi: 10.1039/c8ra10094b (PMC9064204; doi:10.1039/c8ra10094b)
Supplement: RA-009-C8RA10094B-s001 [file RA-009-C8RA10094B-s001.pdf]

Electronic Supplementary Material (ESI) for RSC Advances.
This journal is © The Royal Society of Chemistry 2019

Electronic Supplementary Information

Performance and properties of coking nanofiltration concentrates treatment and membrane fouling mitigation by Fe(II)/persulfate-coagulation-ultrafiltration process

Ming Yang[a], Jiabin Chen[a], Boyu Peng[a], Zhenjiang Yu[a], Huaqiang Chu[a*] and Xuefei Zhou[a*]

[a]State Key Laboratory of Pollution Control and Resources Reuse, School of Environmental Science and Engineering, Tongji University, Shanghai 200092, China.

[*]Corresponding authors: Xue-Fei Zhou (main corresponding author, E-mail: zhouxuefei@tongji.edu.cn; Tel: 86-21-65982693) and Hua-Qiang Chu (chq123wd@163.com).

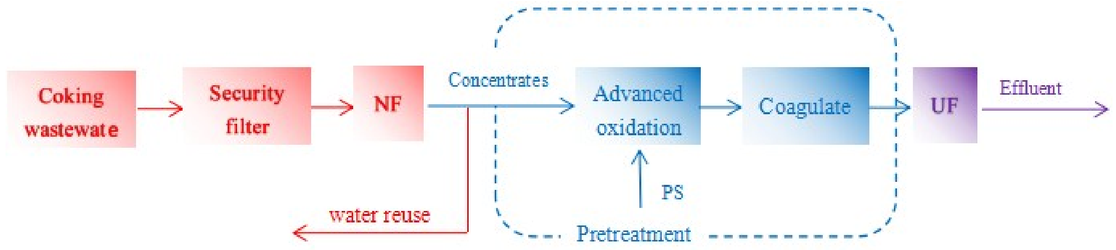

Fig S1: The Fe(II)/PS-PFS pretreatment technology process of coking NF concentrate

Table S1: Characteristics of UF membranes used in the experiments.

Parameter	Specification
Module type	GC-UF0051
pore size	0.05μm
Surface area	66.3m^2
Chemistry	PES
Hydrophobicity [a]	Hydrophilic
Initial flux/permeability (DI water)	800±4.5
L/(m^2·h·bar)	
Zeta potential [b]	-12.4±3.2

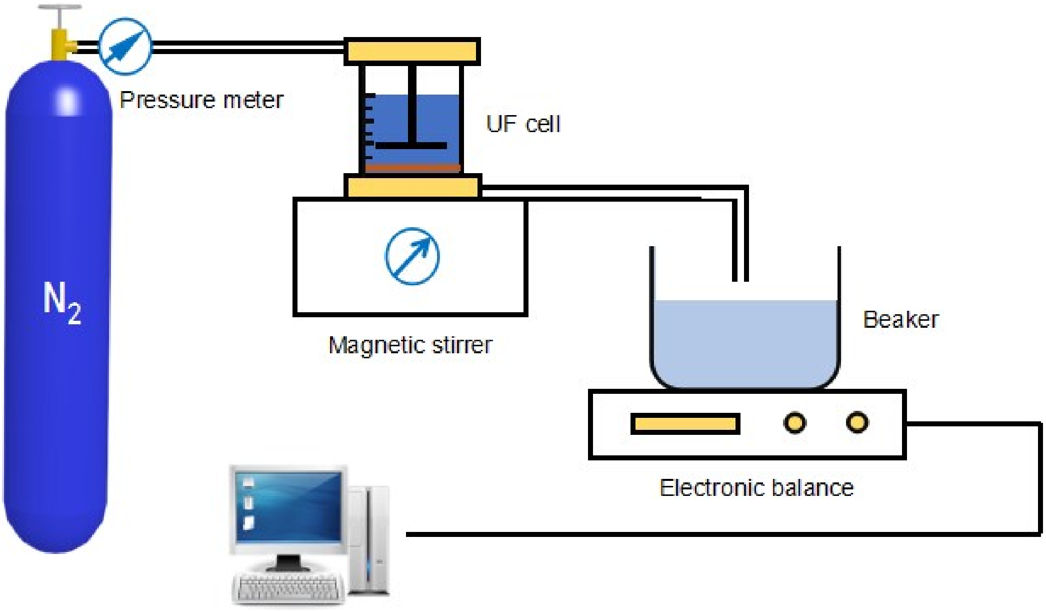

Fig S2: Schematic diagram of the experimental setup.

Table S2: The main organic pollutants by GC×GC-TOFMS

Compound name	Molecular formula	CAS	NF-concentrated water	Oxidized water
			Percentage of integral area	Percentage of integral area
Phenol	C_6H_5OH	108-95-2	28.21	6.33
P-nitrophenol	$C_6H_5NO_3$	100-02-7	10.45	3.12
Chlorine methyl iodide	CH_2Cl_1	593-71-5	9.86	1.32
Indole	C_8H_7N	120-72-9	8.50	0.54
2-methyl-2-butenal.	C_5H_8O	1115-11-3	8.24	2.20
3-methyl-2-butanone.3	$C_5H_{10}O$	563-80-4	4.63	0.80
6-Amino-2-methylquinoline	$C_{10}H_{10}N_2$	65079-19-8	0.89	Nd [a]
1, 2-dichloropropane.	$C_3H_6Cl_2$	78-87-5	0.58	Nd [a]
Naphthalene	$C_{10}H_8$	91-20-3	0.51	0.48
...	...	...	...	...
Total			80.69	14.96

[a] ND refers to not detected.

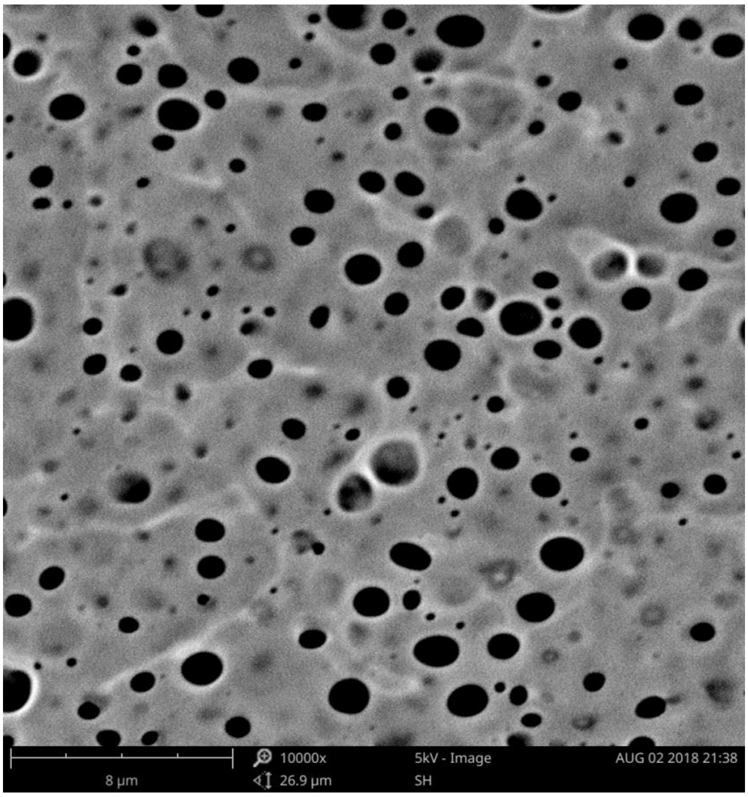

Fig S3: SEM images. virgin UF membrane (10Kx)

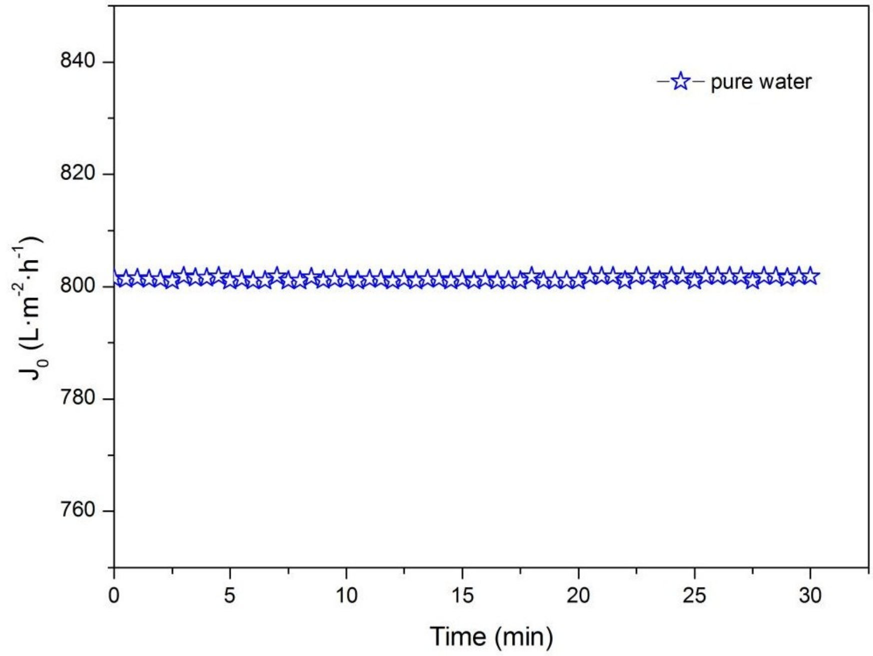

Fig S4: Effects of Fe(II)/PS on the membrane performance. Pure water flux.J_0 represents the initial permeation flux of pure water.

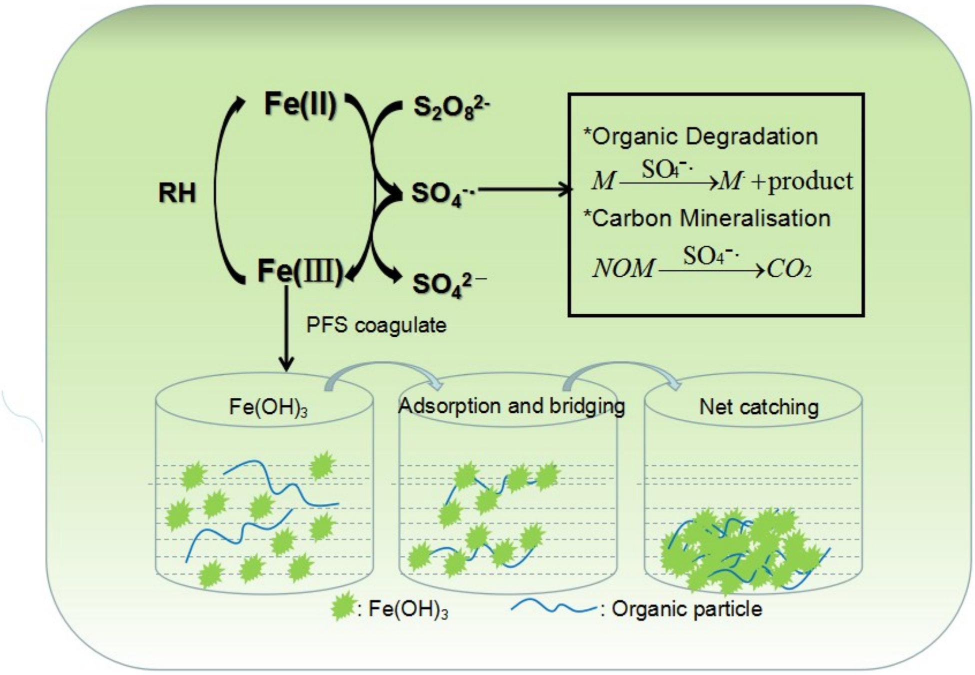

Fig S5: Mechanism of Fe(II)/PS and coagulation.
